# Climate change-related stress and premenstrual symptoms among nursing students: the moderating role of climate change awareness and the mediating role of eco-anxiety

**DOI:** 10.1186/s12912-026-04812-8

**Published:** 2026-06-03

**Authors:** Nagah Abd El-Fattah Mohamed Aly, Safaa M. El-Shanawany, Maha Abdelhamied Ghanem, Elham Mohamed Abd El Kader Fayad, Wael M. Lotfy

**Affiliations:** 1 Nursing Administration, Faculty of Nursing, Matrouh University, Mersa Matrouh, Egypt; 2 Forensic Medicine and Clinical Toxicology, Faculty of Medicine, Alexandria University, Alexandria, Egypt; 3 Psychiatric Nursing and Mental health, Faculty of Nursing, Alexandria University, Alexandria, Egypt; 4 Community Health Nursing, Faculty of Nursing, Matrouh University, Mersa Matrouh, Egypt

**Keywords:** Climate change, Knowledge, Awareness, Stress, Eco-anxiety, Premenstrual syndrome, Nursing, Students

## Abstract

**Background:**

Nursing students are future nurses. They are more susceptible to the physiological and psychological implications of climate change because of their participation in climate activities.

**Aim:**

To assess the relationships among nursing students’ climate change awareness, climate change-related stress and eco-anxiety; and the associations between their climate change-related stress and their eco-anxiety and premenstrual syndrome.

**Methods:**

A cross-sectional correlational study was conducted on a convenience sample of 400 female nursing students at Matrouh University via self-report questionnaires about climate change knowledge and awareness, perceived stress, eco-anxiety and premenstrual syndrome. The data were collected from January 2022 to June 2025. Hierarchical multiple regression analysis was performed to analyze the effects of the mediating and moderating variables.

**Results:**

Nursing students demonstrated satisfactory levels of knowledge and high levels of awareness of climate change. High climate change awareness among nursing students was coupled with moderate (53.2%) to severe (28.6%) climate change-related stress, whereas 54.9% of the students experienced moderate (46.2%) to severe (8.7%) levels of eco-anxiety. Regression analysis revealed that the relationship between nursing students’ climate change-related stress and eco-anxiety was moderated via climate change awareness as a moderating factor. Eco-anxiety played a mediating role in the relationship between climate change-related stress and premenstrual syndrome among nursing students.

**Conclusion:**

Nursing students suffer from mental stress related to climate change, including climate change-related stress and eco-anxiety, due to increased climate change awareness. Climate change-related stress and eco-anxiety increased the prevalence of premenstrual syndrome among nursing students.

## Introduction

Climate change is a significant global challenge and poses an existential threat to the health and well-being of humans [1–3]. Climate change impacts on human health outcomes include increases in mortality and morbidity due to natural disasters and extreme weather events; increases in the frequency and intensity of food-, water-, and vector-borne infectious diseases; the exacerbation of chronic diseases; and increases in mental health problems and stressors [2, 3]. The World Health Organization (WHO) predicts that climate-sensitive diseases will cause approximately 250,000 extra deaths between 2030 and 2050, with the greatest impacts being felt by the most vulnerable populations [4]. 

Climate change is perceived as among the greatest potential threats to women’s health in the twenty-first century [1]. Women are among the most vulnerable population segments in the face of climate change in many areas. Those living in regions with low economic and social status are even more affected by the negative consequences of climate change [5]. Various climate‐related events, such as extreme heat and increased average seasonal temperatures, poor air quality, and natural disasters, have a negative impact on mental health, significantly affect sexual and reproductive health outcomes and increase the mortality rates of women [6, 7]. 

Women may encounter various emotional problems that affect their mental and menstrual health. One of these negative emotions appears to be climate stress and climate change anxiety [5]. Women are more susceptible to the development of climate-related stress, worry and anxiety than men are [6]. Climate change induces a variety of stressors, including extreme weather events, food insecurity, and biodiversity loss. These stressors, coupled with social unrest and uncertainty about the future, can lead to feelings of anxiety, sadness, hopelessness, and helplessness [8]. 

Stress and anxiety resulting from individual and environmental factors cause women to become vulnerable to changes in menstrual cycle length and/or negative changes in symptoms [9–11]. Climate stress and eco-anxiety can influence hormonal fluctuations, neurotransmitter levels, and overall physiological functioning, which can affect premenstrual syndrome (PMS) symptoms. Climate stress and eco-anxiety can affect the hypothalamic‒pituitary‒adrenal axis, which plays a role in regulating the levels of hormones, including those involved in the menstrual cycle. Thus, climate stress and eco-anxiety influence women’s hormonal cycles and are expected to increase the incidence and intensity of premenstrual syndrome (PMS) [12]. 

Similarly, in female nursing students, climatic changes can directly or indirectly lead to a range of mental and menstrual health problems [11, 13]. As nurses, they feel that they have a degree of social responsibility and a professional role in avoiding and minimizing health risks and hazards associated with climate change in community settings. In health care settings, they also play a role in educating patients about the effects of climate change on health [13]. 

Increasing nursing students’ awareness and knowledge of climate change and its consequences can heighten their emotional and cognitive response [1]. They are disproportionately distressed and concerned about climate change, often experiencing strong emotional reactions, even if they have not yet been directly impacted [14]. 

Studies have shown that exposure to climate-related events is associated with greater climate change awareness, knowledge, stress and anxiety [13, 15–17]. Recent research has revealed a complex relationship between stress, anxiety and PMS, with stress and anxiety acting as both risk factors for and consequences of premenstrual symptoms [18–20]. 

In this respect, a well-developed understanding of the associations between female nursing students’ awareness and knowledge of climate change and their mental and menstrual health is critical for developing effective strategies for addressing the consequences of climate change for women’s health. Understanding these associations is also critical for developing effective mental and menstrual health interventions to meet women’s health needs in the context of climate challenges [5, 11]. 

Some studies have focused mainly on the level of awareness of climate change and its association with climate change-related stress and eco-anxiety among nursing students and medical students [13, 15]; however, very little attention has been given to exploring the translation of climate change-related stress into climate change anxiety among nursing and medical students, and they have not focused on climate change awareness as a moderating factor in the relationship between nursing students’ climate change-related stress and eco-anxiety [13, 15]. 

High worries about the environment and eco-anxiety are also associated with more severe PMS [11, 21]. However, there is a paucity of research regarding the effects of mental health problems related to climate change on PMS and women’s health in the literature [11, 21]. 

Although there has been increasing interest in the consequences of climate change for women’s psychological, sexual and reproductive health worldwide and in Egypt, studies exploring the relationships among knowledge about climate change, eco-anxiety and PMS remain limited [11, 21]. Additionally, to our knowledge, no studies examining the relationships among awareness of climate change, climate stress, eco-anxiety and PMS in female nursing students have been conducted. Therefore, the present study aims to address this gap.

## General objective

The present study aimed to assess the relationships among nursing students’ climate change awareness, climate change-related stress and eco-anxiety; and the associations between their climate change-related stress and their eco-anxiety and premenstrual syndrome through the following:

1. Investigating relationships between female nursing students’ awareness and knowledge about climate change and their impact on women’s health, and their climate change-related stress and eco-anxiety.
2. Identifying the relationship between nursing students’ climate change-related stress and their eco-anxiety.
3. Exploring the moderating role of female nursing students’ awareness of climate change in the relationship between their climate change-related stress and eco-anxiety.
4. Identifying relationships between climate change-related stress, eco-anxiety and PMS among female nursing students.
5. Exploring the mediating role of nursing students’ eco-anxiety in the relationships between their climate change-related stress and PMS.

**Operational definitions**:

1. **Climate change-related stress** refers to perceived climate stress and involves a range of negative mental, emotional and physical distress responses to climate change, which are often triggered by climate change events and threats. It can manifest as frustration, anger or nervousness, fear, worry, hopelessness, helplessness and sadness about environmental changes and their future impacts [13, 15].
2. **Eco-anxiety** refers to anxiety related to climate change. It is characterized by chronic fear of environmental doom, persistent worries and continuous stress that stem from climate change threats and the inadequacy of climate action [22–24].
3. **Premenstrual syndrome (PMS)** is a disruptive group of emotional and physical symptoms that regularly occur during the luteal phase of a woman’s menstrual cycle, which is the phase between ovulation and the onset of menstruation [19, 25].

### Theories and hypotheses framework (Fig. 1)

#### H1

Female nursing students’ awareness of and knowledge about climate change and its impact on women’s health are positively associated with their climate change-related stress and eco-anxiety.

Climate change affects human health, including mental health and well-being [26]. The increased occurrence and intensity of extreme weather events, such as heat waves and floods, can produce and intensify mental health stressors [27]. Awareness and knowledge of current climate impacts and impending risks, coupled with perceived inaction, can contribute to the mental and physiological health burden of climate change [27, 28]. 

Awareness and knowledge of climate change can lead to stress and anxiety through direct experience of extreme weather and indirect exposure via media and general concern about the future. Experiencing traumatic events, such as hurricanes, floods and wildfires, and repeatedly seeing or reading about environmental devastation, scientific reports and slow action by environmental institutions cause significant psychological trauma and stressors [29]. As a result of the rising climate crisis, it becomes apparent that the more people feel helpless in their ability to take action, the more they experience a sense of stress, hopelessness and anxiety [30, 31]. 

According to the transactional model of stress and coping and in the context of climate change, exposure to environmental stressors serves as the initiating condition (primary appraisal), which has been hypothesized to increase awareness of climate-related threats. This awareness can influence individuals’ emotional responses (e.g., stress and anxiety) both directly and indirectly through their coping strategies, such as meaning-focused or problem-focused coping [1]. 

Recent studies in different countries revealed that medical and nursing students with greater awareness and knowledge of climate change were more likely to experience psychological distress, climate-related stress, eco-anxiety and hopelessness [32–36]. 

#### H2

Nursing students’ climate change-related stress is positively associated with their eco-anxiety.

#### H3

Female nursing students’ awareness will moderate the relationship between female nursing students’ climate change-related stress and their eco-anxiety, and this relationship will be stronger when their perceptions of climate change are acceptable or high.

The transactional model of stress and coping (1984), appraisal (1990) and analysis of Pihkala (2020) theories provide a helpful framework for understanding how awareness of climate change is associated with climate change-related stress and eco-anxiety. These theories are based on the stress response mechanism [37–39]. 

According to the transactional model of stress and coping theory (1984), stress is a dynamic process arising from the interaction between individuals and their environment. This theory has also been used to understand individuals’ emotional response to climate change and how an individual’s evaluation of a climate stressor affects their coping strategies and ways to respond to it [37]. 

When individuals are exposed to information about climate change, they may perceive it as a significant threat to their well-being, and the environment serves as an initiating condition (primary appraisal), which has been hypothesized to increase awareness of climate-related threats such as climate stressors. They also appraise climate change as a distant threat that reflects a transactional process in which their cognitive evaluation of the situation influences their emotional response (secondary appraisal). This awareness can influence individuals’ emotional responses (e.g., stress) both directly and indirectly through assessing their coping resources and strategies for addressing the perceived threats that lead to mental health stressors known as climate change-related stress. Persistent and continuous climate change-related stress can cause eco-anxiety (reappraisal). These factors contribute to variations in climate change-related stress and eco-anxiety levels [1, 16, 37]. 

According to appraisal theory (1990), stress is viewed as an emotional response to perceived danger or threat. This may imply that individuals may perceive climate change as a significant threat that triggers emotional responses leading to persistent mental health stressors known as climate change-related stress, especially when they are exposed to information about its potential impacts, which may result in heightened eco-anxiety [38]. 

In the context of the theory of analysis of Pihkala (2020) [39], knowledge and awareness of climate change and its consequences can lead to many emotional responses and persistent climate change-related stress, leading to eco-anxiety. Awareness about climate change and its effects can cause a variety of emotions, including guilt, sadness, and anger, all of which contribute to the stress and anxiety associated with climate change. Stress and anxiety can frequently be exacerbated by the constant onslaught of unsettling news about environmental loss and deterioration or the uncertainty of the future, coupled with a sense of hopelessness. Eco-anxiety and climate change-related stress are terms used to describe this condition [16]. 

Recent studies in different countries revealed that medical and nursing students with greater awareness and knowledge of climate change were more likely to experience psychological distress, climate-related stress, eco-anxiety and hopelessness [13, 15, 32–36]. 

#### H4

Female nursing students’ climate change-related stress and eco-anxiety are positively associated with their PMS.

The increased occurrence and intensity of extreme weather events, such as heat waves and floods, can produce and intensify mental health stressors [27]. As a result of climate stress, feelings of worry, sadness, hopelessness and helplessness are exacerbated. These feelings have long been associated with the development of anxiety [8]. 

Climate anxiety is a stressor that can affect one’s behavior, mood, lifestyle, cognitive function, physical health and/or psychological well-being [40]. Hormonal imbalance can also be induced by climate stress and eco-anxiety, which may affect menstrual health.

#### H5

The relationship between female nursing students’ climate change-related stress and PMS is mediated by their eco-anxiety.

Exacerbating climate change-related stress and eco-anxiety due to changes in dopamine and serotonin levels resulting from the effects of extreme heat is more prevalent in women. Women are more susceptible to the development of climate-related stress, worry and anxiety [6, 41]. Numerous studies have highlighted that gender inequality, in particular, exacerbates the severity of the negative effects of climate change on women’s health [30, 31]. Furthermore, previous studies have indicated a correlation between relative humidity and mental health outcomes, including depression, stress, anxiety, and suicide attempts, particularly in women [42, 43]. 

The stress and anxiety associated with climate change can affect PMS both directly and indirectly [44]. Stress induces a hormonal imbalance that regulates the menstrual cycle, leading to disruptions in the menstrual cycle and PMS, affecting fertility and overall well-being [44–46]. This hormonal imbalance can exacerbate PMS symptoms or make them more challenging to manage [26, 47]. 

Therefore, climate change-related stress, along with eco-anxiety, can trigger worsening menstrual symptoms, disrupt menstrual health, be a significant predictor of PMS and potentially exacerbate PMS symptoms for up to two subsequent cycles [45, 46]. A study reported that higher levels of climate change anxiety are linked to a greater prevalence and severity of PMS symptoms. Increasing stress can worsen PMS symptoms and even heighten premenstrual symptoms for up to two subsequent cycles [11]. 

**Fig. 1:**
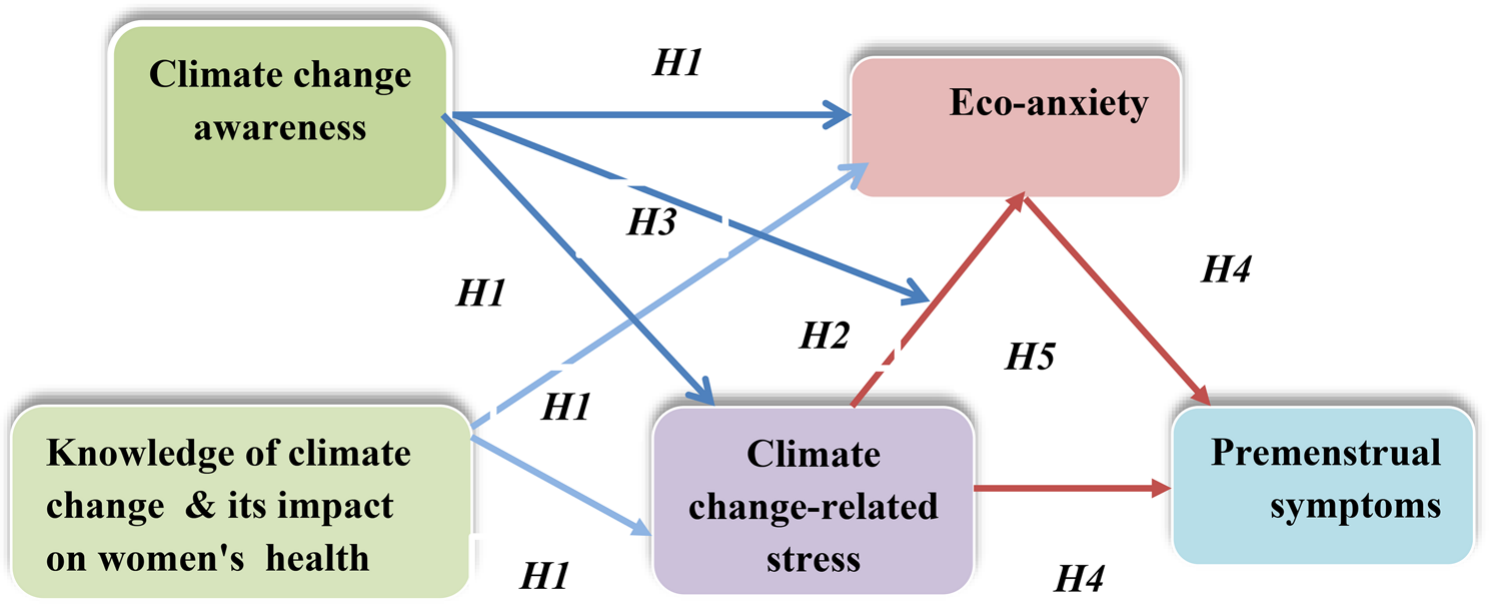
Conceptual framework of the present study

## Materials & methods

### Study settings

This study was carried out at the Faculty of Nursing at Matrouh University, which is located on the Mediterranean coast. Like all coastal cities, coastal cities are exposed to sea level rise, intensified storm surges and coastal erosion and face great challenges in managing the significant growth of climate change exposure.

### Study design

This was a cross-sectional correlational study, and causality cannot be inferred.

### Study sampling and participants

Cochran’s formula (1963) [48] (n = z^2^ p (1-p)/d^2^) was employed to estimate samples via a 5% margin of error (d) and 95% confidence level (Z). According to the students’ registration records at Matrouh University, approximately 50% of the total nursing students (p) registered were females. The minimum required sample size was 385, and the actual sample size was 400 female nursing students.

A convenience sample of female students enrolled from the first academic year to the final academic year and internship students were eligible to participate in the study.

The study sample was chosen on the basis of the inclusion and exclusion criteria. The inclusion criteria consisted of female nursing students who were willing to participate in the study and who signed informed consent forms. The exclusion criteria included female nursing students with diagnosed endocrine disorders, psychiatric conditions and hormone-related medications; students who were unwilling to participate in the study; and those who did not provide informed consent.

## Study tools

1. **Demographic tool**: The control variables used to remove extraneous influences included nursing students’ demographic characteristics, such as age, marital status and place of residence.
2. **Menstrual tool: Menstrual variables** included menstrual characteristics, such as age at menarche, amount, rhythm, duration, interval, and physical and psychological changes before menstruation.
3. **Climate change awareness scale**: This scale was developed by Lopez and Malay (2019) [49] on the basis of Ezeudu (2016) [50] to measure climate change awareness among nursing students. It is composed of 15 items ranked on a five-point Likert scale ranging from strongly disagree (1) to strongly agree (5). The total score was 75 and was classified as very low awareness (15–27 points), low (28–39 points), moderate (40–51 points), high (52–63 points), or extremely high awareness (64–75 points).
4. **Knowledge scale of women’s health impacts on climate change**: Fifteen items were adopted from the studies of Mekawy (2023) [51] (5 items), Saglam & Sahin. (2024) [11] (6 items) and Er et al. (2024) [52] (4 items) to evaluate nursing students’ knowledge of the impact of climate change on women’s health. The answers of the nursing students were between yes (1) and no. (0), and I do not think I am affected (0.5). The total score was 12 points. The nursing students were considered satisfied when the total score was 9 points (≥ 60%).
5. **Perceived Stress Scale (PSS-10)**: The modified PSS-10 was developed by ‏Cohen et al. (1983) [53] and consists of 10 items on a 5-point Likert scale ranging from never (0) to very often (4). The tool was used to measure how often the nursing students experienced feelings of stress or strain related to climate change in the past month.

To estimate the level of perceived stress, the responses of the nursing students to the negative items were scored from never (0) to very often (4), and the responses of the nursing students to the positively phrased items (typically items 4, 5, 7, and 8) were reversed, where “0” became “4,” “1” became “3,” and so forth. These reversed scores, along with the scores of the remaining items, are then summed to calculate the total score. The total scores ranged from 0 to 40 and were classified as follows: (a) low stress (scores ranging from 0 to 13), moderate stress (scores ranging from 14 to 26) and high stress (scores ranging from 27 to 40).

(6). **Eco-anxiety scale**: A 13-point scale was developed by Türkarslan (2023) [54] to evaluate climate anxiety symptoms experienced within the past two weeks‏ among nursing students. Each item is scored on a four-point Likert scale (0 = not at all to 3 = nearly every day). According to Schwaab et al. (2022) [15], scores range from 0 to 39, with higher scores reflecting higher levels of anxiety. The cutoff points for anxiety were 0–7 (minimal), 8–15 (mild anxiety), 16–24 (moderate), and 25 − 9 (severe). Schwaab et al. (2022 [15] scores linked anxiety and climate change and accurately reflected the level of climate anxiety among nursing students in an Egyptian study [13].
(7). **Premenstrual symptoms scale**: A 14-point Likert scale (1 = not at all, 4 = severe) was developed by Choijiljav et al. (2024) [55] to assess the level of premenstrual symptoms in the past one to three months among nursing students from their perspective. The total score ranged between 14 and 56 points and was classified according to Steiner et al.’s criteria (2003) [56] as not having PMS (0–14 points), mild PMS (15–28 points), moderate PMS (29–42 points), or severe PMS (≥ 43 points).

## Validity and reliability measures

To ensure that the study tools were applicable to Egyptian nursing students, psychometric tests were established through validity (face and content validity) and reliability measures (interrater, test-retest and consistency reliabilities). The study tools were assessed by five experts for face and content validation. The experts evaluated the relevance of each item of the study tools. They were asked if all the items were clearly worded and would not be misinterpreted. They were also asked to provide their suggestions and comments for the addition or omission of items.

According to the experts’ comments, no adjustments or modifications were needed, and the study tools were found to be clear and understandable. Agreement and consensus among experts regarding importance, appropriateness and clarity were estimated for interrater reliability via the content validity index (CVI). An average CVI of each item ≥ 0.80 indicated that the questionnaire had high content validity [57]. The study tools were translated into Arabic and back-translated into English by researchers.

Test‒retest reliability was assessed via a pilot study. Forty nursing students (10% of the sample size) were included to ensure the applicability of the study in terms of the understandability and relevance of the items and to estimate the time required to complete the study tools. Forty nursing students completed the same study tools repeatedly at two different times. Those students were later excluded from the study.

Test–retest reliability was assessed after three weeks in the same 40 nursing students. The test-retest reliability was 0.89 for knowledge of women’s health impacts of climate change, 0.84 for climate change awareness, 0.76 for perceived stress, 0.86 for eco-anxiety, and 0.79 for premenstrual symptoms. The internal consistency reliability was evaluated via Cronbach’s alpha coefficient and was 0.89 for the knowledge scale of women’s health impacts from climate change, 0.78 for the perceived stress scale, 0.83 for the climate anxiety scale and 0.95 for the premenstrual symptom scale.

## Data collection

The data were collected from the study nursing students via seven self-report tools. The researchers met the nursing students in lecture rooms to explain the objectives of the study and how to fill out the questionnaires, answer any doubts, and address any queries about the questionnaire and study. The data were collected from January 2022 to June 2025 and from Saturday to Thursday between 9 am and 2 pm. This period was considered a suitable time for the nursing students to complete the study tools. Study tools were distributed among 425 students at the beginning of lectures in the lecture rooms under the guidance of the researchers. The researchers collected the completed questionnaires at the end of the nursing students’ lectures and reviewed each collected questionnaire for completeness. Four hundred completed questionnaires were returned, and the response rate was 94.1%. To increase the response rate, researchers wrote the questionnaire in simple and clear Arabic and provided book notes and pens to increase the response rate among nursing students.

### Statistical analysis

SPSS version 27 was used to analyze the data. The percentage, mean, and standard deviation were employed to describe personal characteristics (demographic and menstrual characteristics) and knowledge variables, dependent variables (premenstrual symptoms), independent variables (climate stress), a mediating variable (climate anxiety) and a moderating variable (climate change awareness). The relationships between the dependent and independent variables and test‒retest reliability were evaluated via Pearson correlation coefficient analyses. Interconsistency reliability was analyzed via Cronbach’s alpha.

Hierarchical multiple regression analysis was used to analyze the effects of the mediating and moderating variables. Thresholds of R^2^ are 0.02 (small), 0.15 (medium), and 0.35 (large). The bootstrapping repeated sampling method was used to sample 5000 times to obtain the path coefficients of the mediation model and ensure the mediating effect. The moderation effect and conditional effects were estimated via the SPSS PROCESS macro v4.3. The interaction effect of the moderating analysis was presented via simple slope analysis and graphical presentation.

The data were analyzed without including control variables in the regression analysis. Nursing students’ demographic characteristics (such as age, marital status, and place of residence) were measured and controlled to avoid the effects of their demographic characteristics on the results. The data were analyzed without including age, marital status, or place of residence in the analysis.

## Results

### Demographic and menstruation characteristics

Among the 400 nursing students, 97.5% were between 19 and 23 years of age, 95.5% were identified as single with no history of previous marriage, and 55.8% resided in urban areas. In addition, the nursing students acquired their information from media platforms (54.2%) and the nursing curriculum (25.0%) (Table 1).

Among the same 400 students, 61.0% were aged at menarche between 13 and 16 years, 68.5% had a regular rhythm for one to five days, 89.0% had a moderate amount of menstruation, and 63.2% had an interval of 25–34 days. A total of 86.5% of the nursing students experienced physical changes before menstruation, whereas 96.5% experienced psychological changes (Table 1).

**Table 1:** Personal and menstrual characteristics of the nursing students (*n* = 400)

Characteristics	No. (%)	Characteristics	No. (%)
		**Amount**	
**Age**:		Scant	16 (4.0)
≤ 18	6 (1.5)	Moderate	356 (89.0)
19–23	390 (97.5)	Heavy	28 (7.0)
≥ 24	4 (1.0)	**Rhythm**	
**Marital status**		Regular	274 (68.5)
Single	382 (95.5)	Irregular	126 (31.5)
Married	14(3.5)	**Interval (days)**	
Divorced	4(1.0)	≤ 24	101 (25.3)
**Place of residence**		25–34	253(63.2)
Urban	223 (55.8)	35–44	26 (6.5)
Rural	177 (44.2)	≥ 45	20 (5.0)
Source of climate change information *		**Duration days**	
Nursing curriculum	100(25.0)	1–5	274 (68.5)
Conference	10 (2.5)	6–10	126(31.5)
Mass media	217 (54.2)	**Physical changes before menstruation**	
Website	73 (18.3)	Yes	346 (86.5)
**Age at menarche (Years)**		No.	54 (13.5)
9–12	112(28.0)	**Psychological changes before menstruation**	
13–16	244 (61.0)	Yes	386 (96.5)
17–20	44 (11.0)	No	14 (3.5)

*Not mutually exclusive

### Descriptive statistics, relationships and comparisons

The nursing students had a high level of climate change awareness (70.5%) and a satisfactory level of knowledge of the impacts of climate change (73.7%) on women’s health. A total of 81.7% of the nursing students were exposed to moderate (53.0%) or high levels of stress (28.7%), with an overall mean score of 26.01 ± 5.43%, whereas 54.9% of the students were exposed to moderate (46.2%) or severe levels of anxiety (8.7%), with an overall mean score of 24.11 ± 6.08%.

Additionally, 83.0% of them exhibited moderate to severe PMS, with an overall mean score of 39.22 ± 4.06. Climate stress was positively correlated with eco-anxiety (*r*=.62, *p* < .01). Climate change awareness was positively correlated with climate stress (*r* = .66, *p* < .01) and eco-anxiety (*r* = .48, *p* < .01). Additionally, knowledge had a significant positive relationship with climate stress (*r* = .64, *p* < .01) and eco-anxiety (*r* = .38, *p* < .01) (Table 2).

**Table 2:** Descriptive analysis and relationships between study variables among the nursing students (*n* = 400)

Climate change	Prevalence/ Level	No. (%)	Scores		Relationships			
			Min–Max	Mean ± SD	1	2	3	4
1- Awareness	Very low	2(0.5)	15–75	52.66 ± 17.82	1	-	-	-
	Low	26 (6.5)						
	Moderate	90 (22.5)						
	High	282 (70.5)						
5- Knowledge	Satisfactory	295 (73.7)	2–15	8.35 ± 2.16	-	1	-	-
	Unsatisfactory	105 (26.3)						
7- Stress	Mild	73(18.3)	6–40	26.01 ± 5.43	0.66**	0.64**	1	-
	Moderate	212 (53.0)						
	Severe	115 (28.7)						
10- Anxiety	Minimal	65(16.3)	0–39	24.11 ± 6.08	0.48**	0.38*	0.62**	1
	Mild	115(28.8)						
	Moderate	185 (46.2)						
	Severe	35 (8.7)						
14- PMS*	Present	332 (83.0)	14–56	39.22 ± 4.06	-	-	0.58**	0.46**
	Absent	68(17.0)						

*Present (moderate to severe), absent (mild or not at all)

** Significant at ≤ 0.05

An independent sample t-test revealed differences between the present PMS group and the absence of PMS groups of nursing students in terms of the mean scores of climate stress (mean ± SD = 26.01 ± 5.43 and 16.64 ± 5.12, respectively; t = 2.881, *p*< .05) and anxiety (mean ± SD= 24.11 ± 6.08 and 18.85 ± 5.20, respectively; t = 6.331, *p*< .05) (Table 3).

**Table 3:** Comparison of nursing students’ climate stress and anxiety between the present and absent PMS groups

PMS	Climate stress (*n* = 400)			Min–Max	Mean ± SD	t test (Sig.)	Climate anxiety (*n* = 400)				Mean ± SD	Min–Max	t test (Sig.)
	Low No. (%)	Moderate No. (%)	Severe No. (%)				Minimal No. (%)	Mild No. (%)	Moderate No. (%)	Severe No. (%)			
Present	17 (2.8)	372 (62.0)	121 (20.2)	6–40	26.01 ± 5.43	2.881 (0.007)*	21(3.5)	27 (4.5)	327(54.5)	139 (23.2)	24.11 ± 6.08	0–39	6.331 (0.000)*
Absent	16 (2.7)	74(12.3)	0(0.0)		16.64 ± 5.12		9 (1.5)	16 (2.7)	41(6.8)	20(3.3)	18.85 ± 5.20		

*p-value was considered significant at ≤ 0.05

### Regression analysis of the moderation effect

The final hierarchical regression analysis in Model 1 revealed a significant impact of climate stress on climate anxiety (Beta = 0.62, *p* ≤ .01). **In Model 2**, climate change awareness added to the model significantly (*β* = 0.38) and predicted eco-anxiety, and this model explained 55.8% of the eco-anxiety.

**Fig. 2:**
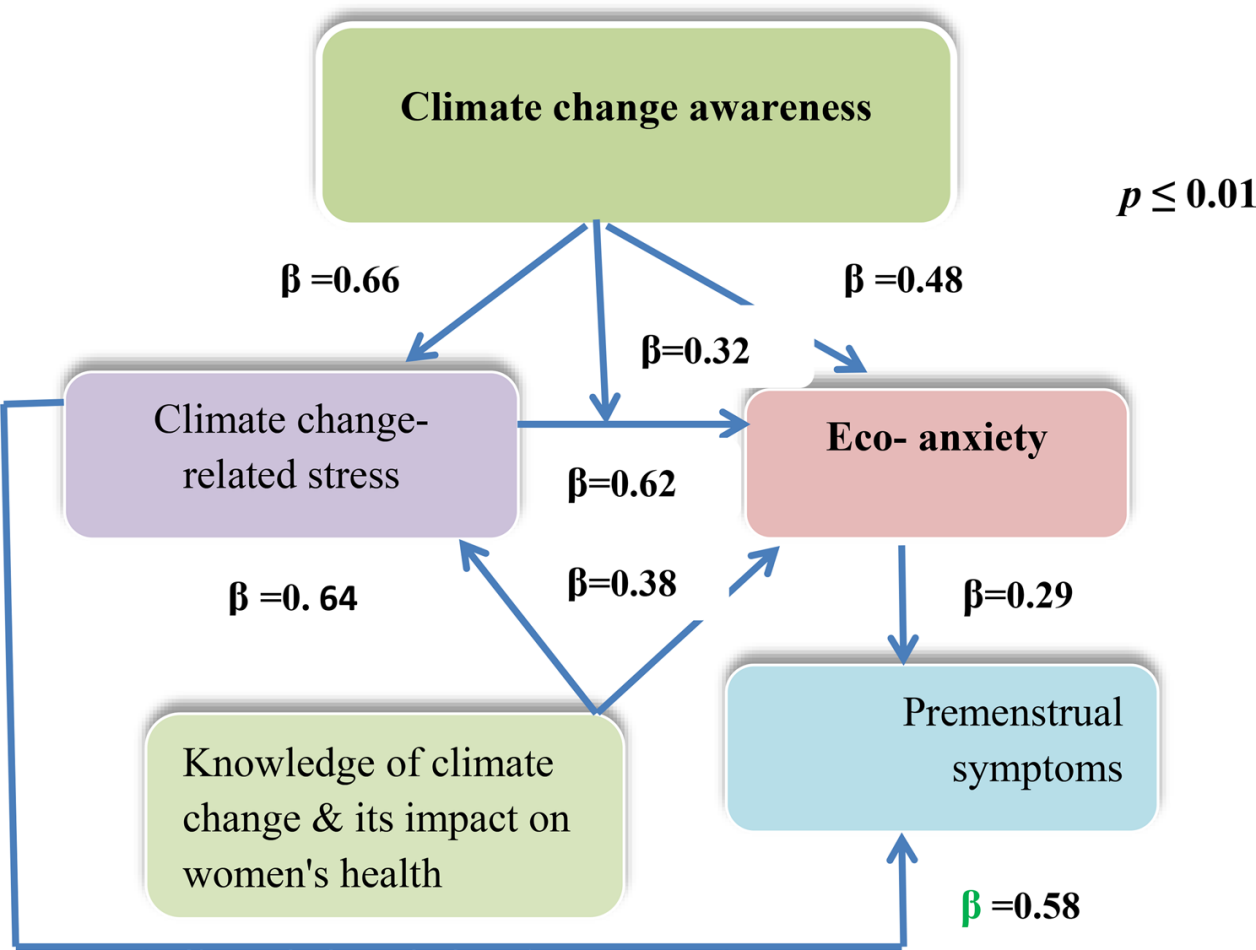
Summaries of the mediating and moderating analyses

**In Model 3**, a positive direct effect of climate change awareness on climate anxiety was found when it interacted with climate stress (Beta = 0.32, *p* ≤ .01) (Table 4). The effects of increasing climate change awareness on eco-anxiety when interacting with climate stress are shown in Model 3 (B=0.29, *R* = 66.3%, F = 40.307, *p* < .01). An increase in R^2^ and a positive statistically significant F test were shown in models 1, 2 and 3 (*R* = 38.9%, F = 83.693, *p* < .01, *R*^*2*^= 55.8%, F = 62.446, *p* < .01 and *R*^*2*^= 66.3%, F = 40.307, *p* < .01, respectively). Therefore, climate change awareness had a positive moderating effect on the correlation between climate stress and anxiety (Table 4; Fig. 2).

**Table 4:** Results of the moderating test

Model	Variables	Climate anxiety				
		B (SE)	Beta	t (sig.)	*R* square	F test (sig.)
1	Climate stress	0.56 (0.10)	0.62**	7.904(0.000)	0.389	83.693 (0.000)**
2	Climate stress	0.50 (0.08)	0.54**	5.113 (0.000)	0.558	62.446(0.000)**
	Climate change awareness	0.38 (0.05)	0.39**	2.797(0.006)		
3	Climate stress	0.57 (0.13)	0.59**	4.302 (0.000)	0.663	40.307 (0.000)**
	Climate change awareness	0.35 (0.09)	. 35**	3.725(0.000)		
	Interaction (Climate stress * Climate change awareness)	0.29 (07)	0.32**	6.073 (0.000)		

Unstandardized Coefficients: B and Std. Error (SE); Path Coefficients: Beta (β) **p values were significant at ≤ 0. 01

### Regression analysis of the mediation effect

Hierarchical regression analyses illustrated in the **first model** in the mediating analysis that climate stress significantly affected eco-anxiety (β = 0.62; *p* **≤** 0. 01) and PMS (β = 0.58; *p* < .01). Climate stress significantly predicted eco-anxiety (B= 0.56, F = 83.693, p **≤** 0. 01) and PMS (B= 0.97, F = 101.149, *p* **≤** 0. 01). Climate stressors accounted for 38.9% of the eco-anxiety symptoms and 33.9% of the premenstrual symptoms (Table 5; Fig. 2).

**In the second model**, when eco-anxiety was added, climate change-related stress significantly increased (β = 0.47; *p* ≤ 0. 01) and predicted PMS (B = 0.79). On the other hand, eco-anxiety significantly (*β* = 0.29; *p* ≤ 0. 01) predicted premenstrual symptoms (B = 0.31, *R*^*2*^ = 41.6%, F = 69.792, *p* ≤ 0. 01), demonstrating that climate change mediated the relationship between climate stress and premenstrual symptoms among female nursing students (Table 5; Fig. 2).

**Table 5:** Mediating effect of climate change anxiety

Model	Variables	Test (1)				Test (2)				
		Anxiety				PMS				
		B	SE	Beta**	t (sig.)	B	SE		Beta**	t (sig.)
1	**Stress**	0.56	0.106	0.62	7.904(0.000)	0.97	0.09		0.58	10.05(0.000)
		R square = 0.389 F test = 83.693**				R square = 0.339 F test = 101.149**				
2	**stress**					0.79		0.09	0.47	8.183 (0.000)
	**anxiety**					0.31		0.06	0.29	5.073 (0.000)
						R square = 0.416 F test = 69.792 **				

Unstandardized Coefficients: B and Std. Error (SE); Path Coefficients: Beta (β) **p values were significant at ≤ 0. 01

### Conditional analysis of the moderation effect

**Fig. 3:**
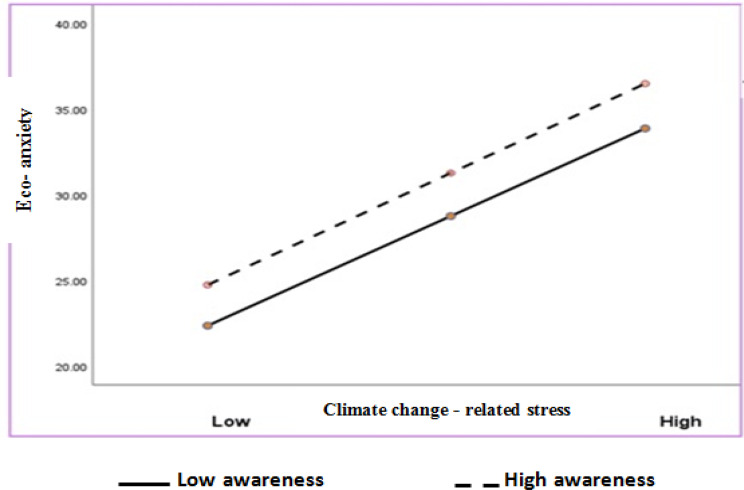
Moderating effect of climate change awareness on the relationship between nursing students’ climate change-related stress and eco-anxiety

The relationship between climate stress and eco-anxiety was positively moderated by nursing students’ climate change awareness. The conditional effect of stress was stronger and significant at a high level of climate change awareness (conditional effect = 0.60 with a 95% CI of 0.44– 0.77, SE =. 08, *t* = 7*.12*, *p* < .05) and was significant at a moderate level of climate change awareness (conditional effect = 0.46 with a 95% CI of 0.25– 0.68, SE = 0.10, *t* = 4:28, *p* < .05). Additionally, it was positive and significant at a low level of climate change awareness (conditional effect = 0.35 with a 95% CI of. 18- 0.51, SE = 0.07, *t* = 3.45, *p* < .05) (Table 6; Fig. 3).

**Table 6:** Moderator effect of climate change awareness

Moderator variable	Moderator level	Effect	SE	t	*p*	95% CI (LL)	95% CI (UL)
Awareness	Low	0.35	0.07	3.45	0.000	0.18	. 51
	Moderate	0.46	0.10	4.28	0.0000	0.25	0.68
	High	0.60	0.08	7.12	0.000	0.44	0.77

Significance at *p* < .05

### Bootstrapping analysis of mediating effects

The results of the bootstrapping procedure with 5000 resamples revealed the indirect and mediating roles of climate change-related stress on PMS through climate anxiety β = 0.31, with a 95% *CI* of 0.18–43, t = 5.073, *p* < .05 excluding 0, whereas the total effect of climate change-related stress on PMS was also found (β = 0.97 *p* < .05). Similarly, climate stress had a significant direct effect on premenstrual symptoms (β = 0.79, *p* <. 05) (Table 7).

**Table 7:** Bootstrapping test of mediating effects

Predictors	Effect		SE	t	Sig.*	BC 95% CI	
	(β)	Sig.*				LLCI	ULCI
Total effect (Stress → PMS)	0.97	0.000	0.08	7.376	**0.000**	0.80	1.14
Direct (Stress → PMS)	0.79	0.000	0.09	8.183	**0.000**	0.61	0.98
Indirect (Stress → anxiety → PMS)	0.31	0.000	0.06	5.073	**0.000**	0.18	0.43

*n* = 400; bootstrap sample size = 5000, BC 9 5% CI= bootstrap confidence intervals, *p < .05

## Discussion

Worldwide, climate change is an environmental factor that affects women in many areas, especially women’s psychological, mental, sexual and reproductive health. Global warming and subsequent climate change significantly increase stress, anxiety and premenstrual disorder levels among women [6, 11, 58]. 

Likewise, the effects of climate concerns are more common among university students. Climate change related to stress and anxiety causes female students to become vulnerable to changes in menstrual cycle length and/or negative changes in symptoms [11, 59, 60]. In this respect, the current study aimed to assess the relationships between climate change awareness, climate-related stress and eco-anxiety and the associations between climate-related stress and eco-anxiety and PMS.

Notably, in the present study, the most of the nursing students were highly aware of climate change and were sufficiently knowledgeable about climate change and its effects on women’s health. A similar finding was also reported in a study conducted in Turkey (2024), which reported that the majority of women were aware of climate change and knew about the effects of climate change on health [11]. The present study was similar to other studies from 2020 to2025 in different countries and concluded that more than half of the undergraduate and nursing students were knowledgeable and aware of the effects of climate change on health [16, 52, 61–64]. 

The sufficient nursing students’ knowledge and high awareness level about climate change and its effect on human and female health in the present study might be related to two main factors. **First**, educational achievements related to climate change and environmental sustainability were strengthened through the use of nursing curricula and conferences. **Second**, the nursing students used different websites, mass media platforms and the internet, which made information easily accessible and increased their knowledge of climate change. Similarly, Bugaj et al. (2021) and Çolak et al. (2025) reported that climate change and health courses, friends, the internet and social media increased students’ awareness levels and acquisition of information about this issue [65, 66]. 

The present study also revealed a positive correlation between nursing students’ knowledge and awareness of climate change and its effects on climate-related stress and eco-anxiety. A high level of climate change awareness and a satisfactory level of knowledge were associated with an increased risk for nursing students suffering from climate change related to stress and eco-anxiety.

In the present study, the nursing students may have experienced heightened levels of climate change-related stress and eco-anxiety because most of them perceived climate change as a threat to their professional role in the future. They feel a sense of responsibility for climate change consequences as future nurses or health team members. This may be because climate change poses significant challenges to the future healthcare environment, potentially affecting both human health and environmental resources.

When nursing students become confused about their professional role as nurses with respect to climate change, they try to acquire more knowledge about their role in managing the threat of climate change to humans. Awareness of climate change has been reported to be a stressful situation that causes high tension, hopelessness and fear, leading to difficulties in coping with stress and increasing the degree of stress experienced by students.

Similarly, other studies reported that frequent and prolonged stressful situations related to climate change in nursing students have led to worsening mental health conditions, such as increased anxiety, irritability and difficulties in concentration. Individuals who were aware of the effects of climate change on human health presented higher levels of climate change-related stress, worry, anxiety and feelings of helplessness [67–70]. 

The findings of the present study align with those of Pihkala (2020) [39], who reported that knowing about climate change and its consequences can lead to many emotions, such as guilt, sadness, and anger, which constitute eco-anxiety. The constant barrage of disturbing news about environmental degradation and loss, or the uncertainty of the future, combined with a sense of hopelessness, can often worsen anxiety.

The findings of the present study were similar to those of two Egyptian studies and other countries, which reported that nursing and medical students with higher perceptions, awareness and knowledge of climate change as a health hazard reported greater levels of climate change-related stress and eco-anxiety because they witnessed the impact of climate change on human health and their nursing profession [13, 15, 22, 32, 36, 63, 65, 66, 71].

However, the findings of the present study contradict those of Ogunbode et al. (2022), who reported that exposure to information about possible solutions to climate change was not associated with climate anxiety, as learning about ways to combat climate threats helps decrease the resulting stress [72]. 

Moreover, the findings of the current work also contrast with those of Schwaab et al. in Germany in 2022, who reported that climate anxiety was significantly less common among medical students [15]. The difference between the present study and the study of Schwaab et al. (2022) [15] could be explained by the fact that it was carried out with medical students in Germany, where they may have learned how to develop personal care, defense abilities and mental health support, especially because the climate and culture of Germany greatly differ from the Egyptian climate and culture. Additionally, this difference can be attributed to differences in education and curriculum between the two countries.

The present study revealed that climate change awareness moderated the relationship between climate change-related stress and eco-anxiety. The increase in climate change awareness was identified as an important moderating factor in the relationship between increased stress and eco-anxiety levels among nursing students. Notably, in the present study, nursing students with high awareness of climate change tended to evaluate situations with relatively high levels of concern, leading to climate change-related stress and eco-anxiety.

This study was supported by studies in India (2014) [73] and Turkey (2023 and 2024) [74, 75], indicating that the climate change awareness of nursing students and nurses was correlated with elevated levels of climate change-related stress, which led to increased levels of environmental anxiety.

The findings of the present study align with the appraisal and transactional model of stress and coping theories, as these nursing students may recognize climate change as a factor that could add excessive burden to their professional role. The appraisal theory suggests that an individual’s emotion is a result of an individual’s evaluation of and response to stressful situations, whereas the transactional model of stress and coping theory posits that stress is a dynamic interaction or transaction between an individual and their environment [16, 37, 38]. 

From the researchers’ point of view in the present study, climate change problems had a considerably more significant emotional and physical influence on nursing students. When nursing students know and become aware of climate change and its consequences for health, they may continuously perceive disruptive news about climate change as a threat to the uncertainty of the effectiveness of coping strategies in the future.

Additionally, excessive student information obtained from the internet as well as the integration of climate change into their curriculum, especially if not associated with knowledge about related defense strategies, might increase their sense of professional responsibility for climate change consequences as future nurses or health team members.

Therefore, increasing awareness of climate change can influence emotional responses, such as guilt, sadness, anger and a sense of hopelessness, which all increase the degree of perceived stress and can often lead to increased anxiety. The present study also revealed a relationship between climate change-related stress and eco-anxiety.

Similar findings were reported in the studies of Bugaj et al. (2021) [65], Ramírez-López (2023) [76] and Mohamed et al. (2025) [13], who reported that when medical and nursing students experienced prolonged perceptions of climate stressors, this perceived stress was translated into eco-anxiety symptoms. Similar results were reported in studies from Nepal (2020) [77] and Egypt (2024) [78], who reported that stressors were among the predictors of depression, anxiety, and burnout among medical students. Nevertheless, the present findings contrast with those of a previous study by Schwaab et al. (2022) [15], which revealed that medical students experienced significant perceived stress related to climate change; however, this stress did not yet manifest as depressive, anxious, or traumatic symptoms [15]. Differences between the two studies were related to the study tools and the type and size of the sample.

Climate change-related stress can lead to changes in the menstrual cycle. These disruptions are often caused by the hormonal response of the body to physical and psychological stressors [9–11]. The current study also revealed that female nursing students with climate change-related stress and eco-anxiety are more likely to have PMS. A significant positive correlation between climate stress and both eco-anxiety levels and the presence of PMS symptoms was found. Similar findings were reported in other studies, which revealed that there was a significant correlation between premenstrual signs and depression, anxiety, and stress disorders [79–84]. 

High levels of stress coupled with increased anxiety can increase the level of cortisol, which affects various hormone levels, leading to a significant effect on menstrual cycle parameters, including ovulatory function [82]. Climate stress and eco-anxiety triggers affect the hypothalamic‒pituitary‒adrenal (HPA) axis to release cortisol, which can disrupt the balance of ovarian hormones involved in menstruation, potentially leading to increased PMS symptoms [82]. 

In the present study, climate change anxiety positively mediated the relationship between climate change-related stress and PMS. Nursing students struggle with stressors from climate change, and increasing levels of their eco-anxiety can cause hormonal disturbances that significantly worsen their PMS. Thus, climate change-related stress and eco-anxiety contributed to the prediction of PMS in the present study.

A similar finding was reported in a study in Turkey, which revealed that eco-anxiety is a significant predictor of PMS among women [11]. The results of the present study were consistent with those of other studies in different countries, which revealed that the prevalence of menstrual cycle irregularity was associated with high levels of perceived stress and anxiety [79–86]. 

The present study revealed that 83% of the nursing students experienced moderate to severe PMS. The prevalence of PMS (moderate to severe) observed in the present study was comparable to that reported in the studies of Abu Alwafa R et al. (2021) [84] (75.4%), Upadhyay et al. (2023) (86%) [87], and Nandakumar et al. (2023) (76.35%) [25]. However, it was lower than that reported in the studies of Shehadeh et al. (2017) (92.3%) [59] and Roomaney and Louren (2020) (92.3%) [60], whereas it was higher than that reported in the studies of Trivedi (2024) [47] (35.3%). This difference might be due to the different diagnostic tools used in the studies, namely, the selected sample, culture and geographic factors [25]. 

### Theoretical implications

The current study provides five key theoretical contributions to climate change and its impact on women’s health, specifically regarding the moderating role of climate change awareness in the relationship between climate change-related stress and eco-anxiety, in addition to the mediating role of eco-anxiety in the relationship between climate change-related stress and PMS among female nursing students.

**First**, this study adds new strengths to the concept that knowing and awareness about climate change and its consequences can lead to many emotions, such as guilt, sadness, and anger, which constitute climate change-related stress and eco-anxiety, according to the theory of analysis of Pihkala (2020) [39]. Awareness and perceptions of current and future climate change-related threats, coupled with insufficient climate action, are associated with a range of psychological and emotional responses, including anxiety, stress, distress, hopelessness, fear, anger, grief, helplessness, frustration, and guilt [27]. The findings of the present study are in line with those of previous studies that concluded that the effects of climate change on human health are associated with increased levels of climate-related stress, worry, anxiety and feelings of helplessness [13, 52, 67–79]. 

**Second**, the present study makes a novel contribution not only by investigating the influence and association between climate change awareness and climate change-related stress and eco-anxiety among nursing students but also by concentrating on how climate change-related stress has translated into climate change anxiety through the moderating role of climate change awareness. Awareness of climate change, coupled with excessive consumption of news, can contribute to increased stress and anxiety about climate change [13, 15]. 

**Third**, this study contributes to the literature concerning the associations between awareness of climate change and climate change-related stress and eco-anxiety within the frameworks of appraisal theory and the transactional model of stress and coping. The current study links these theories to explore how climate change awareness impacts climate change-related stress and eco-anxiety through understanding and emphasizing the dynamic interplay between individuals and their environment in the stress response process (appraising stressors as the first appraisal), assessing coping methods in addressing the threat (secondary appraisal), evaluating climate change threats and continually reevaluating both the stressor and the available resources to address it [6, 50, 51, 58]. Few studies have explored eco-anxiety in light of appraisal theory and the transactional model of stress and coping [1, 16]. 

**Fourth**, this study offers actionable insights for researchers by demonstrating how climate change-related stress influences nursing students’ PMS by testing the mediating effects of climate change anxiety, assisting healthcare providers in advocating for policies that support climate change mitigation and developing climate policy interventions for sexual and reproductive health as keys to improving the lives of women worldwide. When nursing students face heavy pressure and severe climate change stress, they consume their own energy to address their needs effectively. This process is accompanied by a decrease in the ability to cope with stress. Therefore, the continuous perceived pressure and stress of nursing students significantly affects anxiety.

High levels of stress associated with increased anxiety can cause menstrual hormonal disturbances, leading to a significant effect on menstrual cycle parameters, including ovulatory function [82, 89]. One study in Turkey reported that eco-anxiety was a significant predictor of PMS among women [11]. Therefore, this specific topic requires more attention from health policy makers and researchers to understand the global effects of climate change on women’s health and thereby the effects of adverse mental health conditions on women’s PMS, especially young women.

**Fifth**, the study offers direction to health policymakers in incorporating stress management techniques as part of health promotion activities for women. Academic staff and administrators of faculties and universities should also help nursing students release life pressure and stressors related to climate change in academic and clinical settings through the application of stress management and mental health education methods to increase the ability of nursing students to resist pressure and stressors concerning climate change [90]. Therefore, we should implement effective psychological support and counseling for nursing students, improve their mental health status, and help them deal with the threat of various negative emotions [90]. 

### Implications for nursing practice

Nursing students, as nurses in the future, play a crucial role in addressing the consequences of climate change and global warming on women’s health. The present study emphasized the importance of developing strategies aimed at reducing climate change-related stress and eco-anxiety as well as alleviating the resulting PMS symptoms among nursing students. These strategies include the following:

**First**, faculty administrators should consider certain approaches to reduce the climate change-related stress experienced by nursing students, such as incorporating climate literacy, stress management, menstrual health support and mental health support programs into their academic curriculum. These programs could include workshops on stress reduction techniques, the use of stress management, assertiveness skills, time management, counseling sessions, and peer support groups aimed at providing students with coping mechanisms and resources to manage the negative effects of climate change.

Psychoeducational programs may not only contribute to lowering stress and anxiety but also foster a generation of healthcare professionals with increased awareness of climate-related challenges and a positive mindset toward addressing them.

Encouraging climate change education can provide many benefits for nursing students. Integrating climate change education into nursing curricula can equip nursing students with the necessary knowledge and skills to understand and address the health impacts of climate change and the role of nurses in addressing environmental challenges. Educational institutions can empower nursing students to apply environmentally sustainable healthcare to mitigate the effects of climate change on health. Psychological counseling should be provided for nursing students to increase their awareness of sexual reproductive health problems associated with their psychological status.

Courses on the effects of climate change, adaptation, and coping should be added to postgraduate nursing curriculum programs. Postgraduate students specializing in gynecological nursing should receive training aimed at raising awareness about the impact of climate change concerns on women’s psychological, mental, sexual and reproductive health and stress management.

**Second**, university administrators should also provide adequate monetary resources to have educational materials such as pamphlets, posters, slides and models to impart knowledge of women’s psychological, mental, sexual and reproductive health and stress management in faculties.

Climate change education may reduce climate change anxiety among female students and improve their awareness, perceptions, knowledge, behaviors, and policy expectations about climate change. Additionally, creating supportive environments is needed to minimize the burden of climate change on nursing students, with a focus on females.

**Third**, health policy makers should focus on public health practices to reduce nursing students’ climate change-related stress, eco-anxiety and severity of PMS through education and awareness interventions. Platforms such as television and social media could prove beneficial in mitigating the effects of climate change on female nursing students and improving their climate literacy.

## Conclusion

The present work revealed that awareness of climate change plays a moderating role in the relationship between climate change-related stress and eco-anxiety among nursing students. Climate change anxiety plays a mediating role between perceived stressors related to climate change and PMS, which shows that climate change-related stress and eco-anxiety are among the key factors in understanding the prediction of PMS.

The present study concluded that nursing students, as future nurses, should be prepared for topics such as global warming and climate change and their impact on women’s health. To address the impact of climate change on health, nurses must be skilled practitioners, advocates, and change agents.

### Limitations of the study

The present study may be unique in that it aims to assess how climate change awareness affects climate stress and eco-anxiety, in addition to how moderating and mediating factors are related to PMS among nursing students. However, the current study has several limitations, as follows:

**First**, the current study used a cross-sectional design, which hinders the ability to establish causal relationships between determinants and outcomes. Data were collected at a single time point. Therefore, future research should apply longitudinal designs to assess the impact of students’ awareness of climate change on their psychological responses over time. Likewise, future studies can adopt a cohort design. This design also strengthens the research methodology and improves the validity of the results in examining the impact of climate change on psychological response and PMSs. It also allows the observation of longitudinal trends and tracking, the analysis of a defined group of individuals over a designated period and the thorough exploration of connections between variables.

**Second**, all questionnaires were self-reported by the students. This was considered a limitation since the responses, including awareness and knowledge, may change over time. Employing more objective measures could augment the robustness of the findings.

**Third**, despite the large sample size of the study, the study was conducted at a single Egyptian university located in a coastal city. This limits the generalizability of the findings to all nursing students across Egypt. Thus, further large-scale and an in-depth survey are needed to evaluate the wide range of climate-related emotions and experiences. To improve the generalizability of the findings, a more comprehensive understanding of the impacts of climate change on psychological response and PMS should be obtained. Importantly, the manifestations of climate stress, eco-anxiety and PMS may vary across different demographic areas. Future research will need to replicate this study in rural and urban areas where the effects of climate change might be more prominent.

**Fourth**, the use of a convenience sampling procedure may be considered a limitation, as it can introduce sampling bias and reduce the generalizability of the study findings. However, the large sample size may help minimize the impact of individual biases on the overall results and thereby improve the generalizability of the findings.

**Fifth**, although the study used regression analysis to control for confounding variables such as demographic factors, the study was subjected to a lack of control for confounding variables such as general stress, lifestyle factors, hormonal influences, environmental differences or participant behavior. To ensure that the results reflect true causality, the study should involve design techniques (randomized, matching, restriction) and analytical methods (stratification) to isolate the relationships between independent and dependent variables.

### Future research

**First**, stress management has an important goal of psychological intervention in health emergencies to address the effects of climate change on mental health. Therefore, future research needs to explore the effects of stress management on alleviating the mental health impacts of climate change and PMS among nursing students.

**Second**, nursing students, as future nurses, should be prepared to reduce the consequences of climate change for human health and to be ready to carry out their role in the protection of public health in the face of climate change. Future studies should assess the environmental literacy levels of nursing students as future nurses on global climate change and its impact. Future research should also aim to increase the knowledge and skills of nursing students with respect to their professional role responsibility as nurses with respect to climate change.

## Data Availability

All generated and analyzed data in this research are included in this manuscript. The datasets used and/or analyzed during the current study are available from the corresponding author upon reasonable request.
